# Prophylactic and curative effects of *Carica papaya Linn*. pulp extract against carbon tetrachloride-induced hepatotoxicity in male rats

**DOI:** 10.1007/s11356-022-24083-5

**Published:** 2022-11-17

**Authors:** Nadia Zaki Shaban, Olfat M. Awad, Ghada M. Fouad, Afaf M. Hafez, Ahmed Alaa Abdul-Aziz, Sarah M. El-Kot

**Affiliations:** 1 Department of Biochemistry, Faculty of Science, Alexandria University, Alexandria, 21568 Egypt; 2 Department of Histology and Cell Biology, Faculty of Medicine, Alexandria University, Alexandria, 21563 Egypt; 3 Department of Environmental Studies, Institute of Graduate Studies and Research, Alexandria University, Alexandria, 21526 Egypt; 4 Endocrinology Unit, Department of Internal Medicine, Faculty of Medicine, Alexandria University, Alexandria, 21563 Egypt

**Keywords:** *Carica papaya Linn.* pulp extract, CCl_4_, Oxidative stress, Inflammation, Fibrosis, Apoptosis, Rat liver injury

## Abstract

Several chemicals and medications induce cellular damage in various organs of the body by activating reactive substances’ metabolism leading to various pathological conditions including liver disease. In this study, we evaluated the prophylactic and curative effects of *Carica papaya Linn*. pulp water extract (PE) against CCl_4_-induced rat hepatotoxicity. Five groups of rats were created, control, PE, CCl_4_, (PE-CCl_4_): The rats were administered with PE pre and during CCl_4_ injection, and (PE-CCl_4_-PE): The rats were administered with PE pre, during, and after CCl_4_. The markers of oxidative stress (“OS”: oxidant and antioxidants), inflammation [nuclear factor-κB, tumor necrosis factor-α, and interleukin-6], fibrosis [transforming growth factor-β], and apoptosis [tumor suppressor gene (p53)] were evaluated. Additionally, liver functions, liver histology, and kidney functions were measured. Also, PE characterization was studied. The results showed that PE, in vitro, has a high antioxidant capacity because of the existence of phenolics, flavonoids, tannins, terpenoids, and minerals. Otherwise, the PE administration [groups (PE-CCl_4_) and (PE-CCl_4_-PE)] exhibited its prophylactic and therapeutic role versus the hepatotoxicity induced by CCl_4_ where PE treatment improved liver functions, liver histopathology, and renal functions by decreasing oxidative stress, inflammation, fibrosis, and apoptosis induced by CCl_4_. Our study elucidated that PE contains high amounts of phenolics, flavonoids, tannins, terpenoids, and ascorbic acid. So, PE exerted significant prophylactic and curative effects against hepatotoxicity induced by CCl_4_. These were done by enhancing the markers of antioxidants and drug-metabolizing enzymes with reductions in lipid peroxidation, inflammation, fibrosis, and apoptosis. PE administration for healthful rats for 12 weeks had no negative impacts. Consequently, PE is a promising agent for the prohibition and therapy of the toxicity caused by xenobiotics.

## Introduction

People are constantly exposed exogenously to different amounts of chemicals. These chemicals have been revealed to have mutagenic or carcinogenic properties in experimental frameworks. Exposure can happen exogenously when these chemicals are present in air, food, or water, and endogenously when they are metabolized and yield pathophysiological states such as inflammation. Toxicants are artificial toxic chemicals, and they could be created by humans or occur naturally (Manahan 2009). Alternatively, toxins are poisons produced in the living cells or organs of animals, insects, plants, and bacteria (Manahan 2009; Hodgson 2011). Toxicants (xenobiotics) are characterized by vast production and distribution processes, and increasing ubiquity in the environments, homes, and bodies. Toxicants can be present in various forms in the air, water, food, and soil (Manahan 2009; Hodgson 2011). Toxicants are processed in the human body through enzyme-catalyzed phase I and phase II processes. Lipophilic xenobiotic substances are prone to phase I reactions, which make them more water-soluble and interact through polar functional group correlation. The cytochrome P-450 enzymatic system catalyzes the majority of phase I operations, which are microsomal mixed-function oxidase reactions. Conjugated reactions are referred to as phase II reactions. It happens when an endogenous type is related to the activity of an enzyme on a polar functional group, which is usually the result of phase I xenobiotic reactions. The product of the conjugation of the phase II reactions is usually less soluble in lipids, more soluble in water, less toxic than the original xenobiotic compound, and easier to eliminate from the body (Chen 2012). In addition to the cellular response to cytokines, bacterial invasion, and xenobiotics, reactive oxygen species (ROS) are produced by mitochondrial oxidative metabolism. The imbalance caused by overflowing ROS or oxidants over the cell’s ability to develop an effective antioxidant response is referred to as oxidative stress (OS) (Shaban et al. 2021c). Various disease conditions, such as diabetes, atherosclerosis, neurodegeneration, and cancer, are linked to the OS, which causes macromolecular damage (Diao et al. 2011; Lixin et al. 2019). The oxidation of cysteine residues on proteins by ROS changes protein structure and/or function. When cysteine residues are oxidized, reactive sulfenic acid is formed, which can form disulfide bonds with nearby cysteines or be further oxidized to sulfinic or sulfonic acid. Sulfenic acid can also be transformed into sulfenamide in the existence of nitrogen. These redox alterations can be reversed by reducing systems like glutathione (GSH), and thioredoxin, except sulfonic acid, and to a lesser extent sulfinic acid (Roos and Messens 2011). The antioxidants play a remarkable function in the antagonizing and quenching of free radicals to obtain an equilibrium among free radicals and the antioxidants for normal physiological function. If the equilibrium is skewed towards free radicals, a variety of pathological diseases develop (Shaban et al. 2021a, b, c). Antioxidants have anti-inflammatory, anti-allergic, antithrombotic, antiviral, and anti-carcinogenic properties in addition to their ability to eliminate free radicals.

CCl_4_ is a synthetic chemical and does not occur naturally in the environment. It is a powerful hepatotoxic chemical that is commonly used to cause hepatic fibrosis/cirrhosis, hepatocellular cancer, and liver damage in experimental animals (Reyes-Gordillo et al. 2017; Shaban et al. 2021a, b, c, 2022a). Nevertheless, it has many industrial applications. It was primarily used to make chlorofluorocarbons used in refrigeration. In addition, it was employed as a cleaning agent and a component in fire extinguishers (Reyes-Gordillo et al. 2017; Abu-Serie et al. 2021; Shaban et al. 2021a, b, c, 2022a). Because of the health risks and the substantial environmental harm caused by chlorofluorocarbons, its usage has been phased out by advanced various nations. But till now, CCl4 is used to show watermarks on stamps, and it is employed as a chlorine source according to the Appel reaction. CCl_4_ has been utilized in proton NMR spectroscopy. Also, CCl_4_ is utilized in the production of lava lamps. The oxidative damage caused by CCl_4_ in tissues can be explained as lipid peroxidation where lipid peroxidation starts after activation of CCl_4_ by cytochrome (CYP) 2E1, CYP2B1, or CYP2B2, and possibly CYP3A, forming the trichloromethyl radical CCl_3_*. Oxygen reacts with CCl_3_* to form the trichloromethyl peroxyl radical, CCl_3_OO*, a highly reactive. CCl_3_OO* starts the lipid peroxidation chain reaction, which targets and degrades polyunsaturated fatty acids found in phospholipids (Reyes-Gordillo et al. 2017; Shaban et al. 2021a, b, c, 2022a).

According to recent studies, antioxidants derived from natural sources are an effective strategy to prevent or eliminate the detrimental effects caused by hazardous substances or medications (Shaban et al. 2013, 2022b; Nisar et al. 2017). In comparison to manufactured medications, antioxidants include a lot of phenol chemicals and have fewer negative effects (Muhammad et al. 2019; Abu-Serie and Habashy 2020; Shaban et al. 2020, 2021a, b, c). *Carica papaya Linn.* (*C. papaya*), a tropical fruit, is widespread around the world and present in yellow-green, yellow-orange, and orange-red colors (Malacrida et al. 2011; Shaban et al. 2021a, b, c). *C. papaya* pulp has a high nutritive value. The ripened papaya pulp is commonly eaten fresh like a melon, just peeled and seedless. It is used in the food industry such as marmalade, puree, jelly, jam, ice cream, juice, chunks, mixed beverages, and papaya powder (Saran and Choudhary 2013).

The papaya pulp is rich in minerals and vitamins, especially A, B, C, and K (Hassan et al. 2013). Also, papaya pulp contains flavonoids and alkaloids such as carpasemine and carpain (Hassan et al. 2013). The quantities of flavonoids in papaya pulp are impacted by the fruit’s ripeness (Addai et al. 2013). Danielone, a phytoalexin substance, is specific to papaya fruit and is responsible for the antifungal activity of the plant against several fungal types (Colletotriclum and Gloesporioides) that affect papaya. Additionally, it has been shown that the papaya pulp prevents heart attacks and strokes. The unripe papaya pulp contains various types of digestive enzymes such as papain and chymopapain (i.e., vegetable pepsin) which help in the digestion of food proteins. Otherwise, the unripe papaya fruits contain latex content, so it is never eaten. Consequently, the current study was designed to investigate the prophylactic and therapeutic effects of *C. papaya pulp* extract (PE) against CCl_4_-induced hepatotoxicity where we predestined the antioxidant and anti-inflammatory, antiapoptotic, and antifibrotic impacts of PE via the determination of their indicators. Also, the liver functions, lipid profile, kidney functions, and histological examination of the liver were determined. The phytochemical constituents and characterization of PE were evaluated.

## Materials and methods

### Chemicals and reagents

Rutin (RU), gallic acid (GA), catechin, ursolic acid (UA), Folin–Ciocalteau reagent, 2,2 diphenyl-1-picrylhydrazyl (DPPH), 2,4 dinitrophenyl hydrazine (DNPH), 5, 5′, dithiobis-2-nitrobenzoic acid (DTNB), butylated hydroxytoluene (BHT), CCl_4_ (reagent grade, 99.9%), 2,2-azinobis (3-ethylbenzothiazoline-6-sulfonic acid) (ABTS), NADPH, and GSH were acquired from Sigma-Aldrich, St Louis, MO, USA. Thiobarbituric acid (TBA) was gained from El-Nasr Pharmaceutical Chemicals Co. (Alex., Egypt). Ascorbic acid [(Asc): vitamin C] and Trolox were bought from Riedel-de Haën, Germany. Biozol reagent was purchased from Invitrogen, CA, USA. SYBER Green 1-step qRT-PCR Kit was purchased from Thermo Scientific, USA. Primers for tumor necrosis factor (TNF)-α, nuclear factor-kappa B (NF-κB), transforming growth factor (TGF)-β1, interleukin (IL)-6, and the tumor suppressor gene p53 were acquired from Bioneer, Korea. Kits for alanine aminotransferase (ALT), aspartate aminotransferase (AST), alkaline phosphatase (ALP), total protein (TP), albumin, creatinine, urea, triglycerides (TG), low-density lipoprotein cholesterol (LDL-c), and high-density lipoprotein cholesterol (HDL-c) were gained from Biodiagnostic, Cairo, Egypt.

### Plant

The Caricaceae family’s *C. papaya* fruit was obtained from Nubaria, Behera, Egypt. Fruits were chosen based on shape, regularity, color, size, and the absence of fungal disease.

### Preparation of PE aqueous extract

The peel and seeds from the unripe fresh fruit pulp were removed. About 500 g of the fruit flesh was cut into pieces and homogenized with 0.5 L of distal H_2_O using a blender (Moulinex, France). The homogenate was filtered using gauze and the filtrate was lyophilized using Lyophilizer (Virtis 248625 Freeze Dryer; USA), where the residue was stored in a sealed bottle at 4 °C for further studies (Josiah et al. 2011).

### Characterization of PE

#### Resolve the total phenolics, flavonoids, tannin, triterpenoid, and Asc

The total phenolic content was established as GA equivalents (eq) in mg/g PE, employing the Folin–Ciocalteau reagent (Taga et al. 1984). The total flavonoid content was measured as mg rutin eq/g PE, applying 5% sodium nitrite solutions and 10% aluminum chloride (Zhishen et al. 1999). Also, total tannin content was assessed calorimetrically like mg catechin eq/g PE, employing 2% vanillin in methanol (Price et al. 1978). The content of triterpenoid was defined as mg ursolic acid eq/g PE by utilizing 5% vanillin in glacial acetic acid (Bai et al. 2007). Moreover, the concentration of Asc in PE was assessed by applying 2,4 dinitrophenyl hydrazine (Omaye and Reddy 1962).

#### Phenolics and flavonoids assessment

High-performance liquid chromatography (HPLC) was utilized for the separation of PE (100 µL) by employing a chromatographic column 5 μm, 4.6 mm × 150 mm Eclipse XDB–C18 (Agilent Technologies, Palo Alto, CA, USA), where the divorce flow rate was put at 0.75 mL/min, wavelength 320 nm, and the mobile phase employed was acetonitrile: 1% formic acid: 2-propanol (22:70:8), pH 2.5 Zhu et al. (2004).

#### Minerals assessment

One gram of fresh papaya pulp was put in a porcelain crucible and ignited in a muffle furnace at 500 °C for 12 h to obtain ash. The ash was cooled, dissolved in 5 mL nitric acid (6 M), warmed, and filtrated using acid-washed filter paper. The filtrate was diluted using deionized H_2_O to 25 mL and then the mineral contents, including Ca, Co, Cu, Fe, K, Mg, Mn, Mo, Na, Ni, S, Se, and Zn, were determined employing an inductively 5100 coupled plasma optical emission spectrometer (ICP-OES, AGILENT, USA) (Alzahrani et al. 2017).

### Total antioxidant capacities (TAC) of PE

TAC of PE as well as Asc (as standard) was determined according to the method of Tyagi et al. (2010). The scavenging activity of PE and Asc against ABTS + was detected using Trolox as standard (Re et al. 1999). The ferric-reducing power of the PE and Asc was measured as claimed by Tyagi et al. (2010). Also, the DPPH scavenging capacity of PE and Asc was evaluated according to the method of Blois (1958) with some improvement. Briefly, 100 µL of DPPH was added to 500 µL of successive concentrations (0–1 mg/mL) of PE, ethanol (as control), and Asc (as standard), mixed well, and incubated in the dark for 20 min and at 25 °C, and then the absorbances were recorded at 490 nm. The scavenging activity of PE and Asc against DPPH was measured consistent with the following equation:

$$Inhibition \left(\%\right)=[1-({Absorbance}_{\mathrm{extract}}/{Absorbance}_{\mathrm{control}})\times 100]$$

The relationship between the inhibition (%) and different concentrations of PE and Asc was plotted to estimate their IC_50_ (50% inhibitory concentration) (Blois 1958).

### The biological impact of PE on hepatotoxicity caused by CCl_4_

#### Animals

Forty mature male Sprague–Dawley rats (8–10 weeks old and 150 ± 20 g) were purchased from the Faculty of Agriculture, University of Alexandria, Egypt. The rats were verified for normal health condition and were maintained for 2 weeks for laboratory environment adaptation in well-ventilated cages, an ambient temperature: 25 ± 0.5 °C, and a 12-h light/dark cycle. The rats were given free access to standard commercial rat food and tap water (Shaban et al. 2021a, b, c).

#### Experimental strategy

**Fig. 1:**
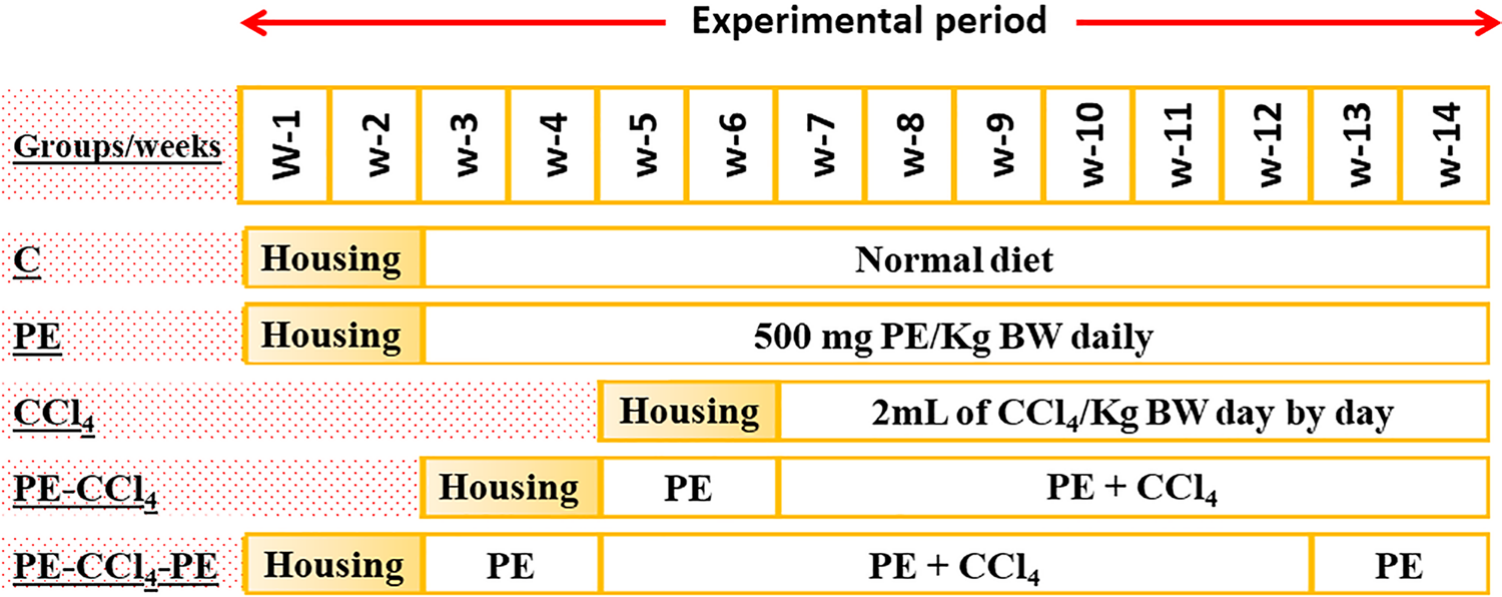
The experimental design

All animals were distributed into 5 groups, 8 rats per each. Group 1 (C): in the control rats without any treatment (*n* = 8); group 2 (PE): rats were orally administered (utilizing oral gavage) with 500 mg of PE/kg body weight (BW) daily for 12 weeks (Josiah et al. 2011); group 3 (CCl_4_): in the intoxicated group (*n* = 8), the animals were subcutaneously (s.c.) injected with 2 mL of CCl_4_/kg BW for 8 weeks (3 times/week) (Karabulut et al. 2014); group 4 (PE-CCl_4_): in this group, rats were treated with PE (the same as in group 2) and at the start of the 3rd week, they were injected (s.c.) with CCl_4_; and group 5 (PE-CCl_4_-PE): rats were treated with PE and CCl_4_ like in group 4 with continual treatment with PE for 2 weeks following the end of CCl_4_ injection. The experimental design is shown in Fig. 1.

At the completion of the experiment, feeding was clogged 12 h prior to scarification. Carbon dioxide gas was used to anesthetize rats for dissection. The blood was gathered from the portal veins in clear test tubes, allowing clotting by standing for 15 min at room temperature, and centrifuged for 10 min and at 1000 × g and the serum was kept at − 20 °C till used for the determination of liver functions, lipid profile, and kidney functions. The liver’s specimens were isolated, soaked in cold saline solution (0.9% NaCl), and split immediately into three parts. The first one was placed in formalin (10%) for the histological examination. The second portion was saved at − 80 °C in RNA later solution till used for the determination of markers of inflammation, apoptosis, and fibrosis. The third sample was homogenized in cold sodium phosphate buffer (0.1 M; pH 7.4) comprising saline solution, centrifuged for 20 min at 10,000 × g and the supernatant was maintained at − 80 °C till used for the determination OS parameters (Shaban et al. 2021a, b, c).

#### Biochemical assays

##### Lipid profile and liver and kidney function tests

For assessment of liver function, the TP and albumin levels and the activities of ALT, AST, and ALP were determined using kits (Gornall et al. 1949; Doumas et al. 1971; Reitman and Frankel 1957; Belfield and Goldberg 1971). Also, the lipid profile (TG, LDL-c, and HDL-c) was evaluated in the serum using kits (Richmond 1973; Fassati and Prencipe 1982). Kidney functions including creatinine and urea levels were assayed using kits (Patton and Croush 1977; Jaffé 1986).

##### Determinations of OS markers

The oxidants [malondialdehyde (MDA) and nitric acid (NO)] were determined in liver homogenates for the estimation of lipid peroxidation and protein oxidation, respectively. MDA was analyzed using TBA (Ohkawa et al. 1979). The NO level was evaluated using the Griess reagent (Montgomery and Dymock 1961). The antioxidant parameters were estimated in the liver homogenates. The GSH level was evaluated according to Ellman (1959) by the reaction of GSH with DTNB providing a yellow product measured at 412 nm and expressed as mg/mg protein. Glutathion reductase (GSR) activity was determined according to Goldberg and Spooner (1983) by the oxidation of NADPH in the presence of GSSG (oxidized form of GSH) and the product was measured at 340 nm, expressed as µmol/min/mg protein. The glutathione-S-transferase (GST) activity was evaluated by the reaction of GSH with GST substrate (p-nitrobenzyl chloride) forming a product which measured at 310 nm (Habig et al. 1974). The superoxide dismutase (SOD) activity was established by an indirect process (Marklund and Marklund 1974). The activity of SOD is characterized as the enzyme quantity that inhibits the pyrogallol autoxidation rate throughout standard conditions, and the variation in the absorbance at 420 nm was determined in 2 min. SOD is expressed as U/mg protein. The activity of total glutathione peroxidase (t-GPx) was measured by establishing the oxidation of NADPH in the sample in the existence of cumene hydroperoxide and GSH at 412 nm (Paglia and Valentine 1967).

##### Determination of markers of inflammation, fibrosis, and apoptosis

Assessment of the expression of NF-κB, IL-6, TNF-α, TGF-β, and p53.

The hepatic RNA of each rat was extracted in accordance with the kit’s instructions. The frozen liver tissues were cut into small slices, transferred to an Eppendorf tube containing 1 mL Biozol reagent (Mou et al. 2013) and then the tissues were homogenized using a glass homogenizer. The homogenate was incubated at 4 °C for 15 min, then 1 mL glycogen was added and combined well, next chloroform was added, and the mixture was left for 15 min and at 4 °C. For the precipitation of the RNA content, the mixture was centrifuged and the aqueous layer was assigned into nuclease-free Eppendorf tube and the same volume of cold isopropyl alcohol was added. The precipitate (RNAs) was then washed, processed with DNAase to remove any remaining DNA, and held at − 80 °C until utilized. The absorbance of RNA samples at 260 and 280 nm ratio (A260/A280) was used to assess the quality of the extracted RNA samples. A spectrophotometer (BioDrop Lite, Australia) was used to determine the amount of RNA, and gel electrophoresis on 2% agarose gel stained with ethidium bromide was used to confirm the quality of the RNA.

Quantitative reverse transcriptase PCR was used to measure the levels of NF-κB, IL-6, TNF-α, TGF-β, and p53 expressions in the extracted RNA samples using a SYBR green PCR master mix one-step kit (Todorova et al. 2006; Shimojo et al. 2006; Chiu and Yang 2007; Yar et al. 2011; Róka et al. 2019). In brief, in a 10 μL reaction volume, the following ingredients were added in the following order: 0.5–3.4 μL RNA template (RNA sample), 5 μL 1-step QPCR SYBER mix (1 ×), 0.5 μL of each forward and reverse primers, 0.5 μL RT-enhancer, 0.1 μL verso enzyme mix, and 0–2.9 μL water (PCR grade) and at 95 °C for denaturation. The reactions included one cycle 10-min reverse transcription at 45 °C, one cycle 2 min of polymerase activation at 95 °C, and tracked by 40 cycles for 15 s and at 95 °C for denaturation, then annealing for 1 min and at 60 °C and the extension for 30 s and at 72 °C. Based on the number of PCR cycles where the increasing fluorescence curve crosses a threshold cycle, the expression levels of all groups under study were determined (CT). The relative expressions of NF-κB, IL-6, TNF-α, TGF-β, and p53 genes were achieved applying comparative CT (ΔΔCT) method, and β-actin (reference gene) was used as internal control. ΔCT and ΔΔCT were calculated by the following equations: ΔCT = CT (any marker) − CT (β-actin) and ΔΔCT = ΔCT (Sample) − ΔCT (β-actin control). The expression fold changes were calculated from this formula: Expression fold change = 2 − ΔΔCT (Shaban et al. 2022a, b). All primers were used are recorded in (Table 1).

**Table 1:** Quantitative reverse transcriptase polymerase chain reaction (RT-PCR) technique

Name	Sequence	Product size	Accession number
Beta-actin	Forward: AGC CAT GTA CGT AGC CAT CC	189	NM_031144
	Reverse: CTC TCA GCT GTG GTG GTG AA		
P53	Forward: GTC GGC TCC GAC TAT ACC ACT ATC	246	NM_030989
	Reverse: CTC TCT TTG CAC TCC CTG GGG		
NF-κB	Forward: ACG ATC TGT TTC CCC TCA TCT	154	AF079314.2
	Reverse: TGC TTC TCT CCC CAG GAA TA		
IL-6	Forward: AGT TGC CTT CTT GGG ACT GA	217	M26744
	Reverse: ACA GTG CAT CAT CGC TGT TC		
TGF-β	Forward: CTT TGC TCA TGG CAG TAC ATC TG	152	NM_013174
	Reverse: CCT TTA ACA ACA TCC CGA TTC C		
TNF-α	Forward: AGA TGT GGA ACT GGC AGA GG	178	X66539
	Reverse: CCC ATT TGG GAA CTT CTC CT		

##### Histological probation of liver tissues

For liver histological investigations, the liver tissues were cleaned, mended, and encased in paraffin wax (Suzuki and Suzuki 1998). Hematoxylin and eosin (H&E) stain was used to stain sections of 5 μm thickness.

### Statistical analysis

Comparative analyses matching between the means of the two groups were performed using SPSS software to examine the antioxidant and anti-inflammatory properties of PE against CCl_4_-induced liver damage (Version 25). The data, which was provided as a mean standard deviation, were analyzed using one-way ANOVA analysis (SD). The significance threshold was set at *p* < 0.05.

## Results

### PE description

#### PE mineral and phytochemical compositions

**Fig. 2:**
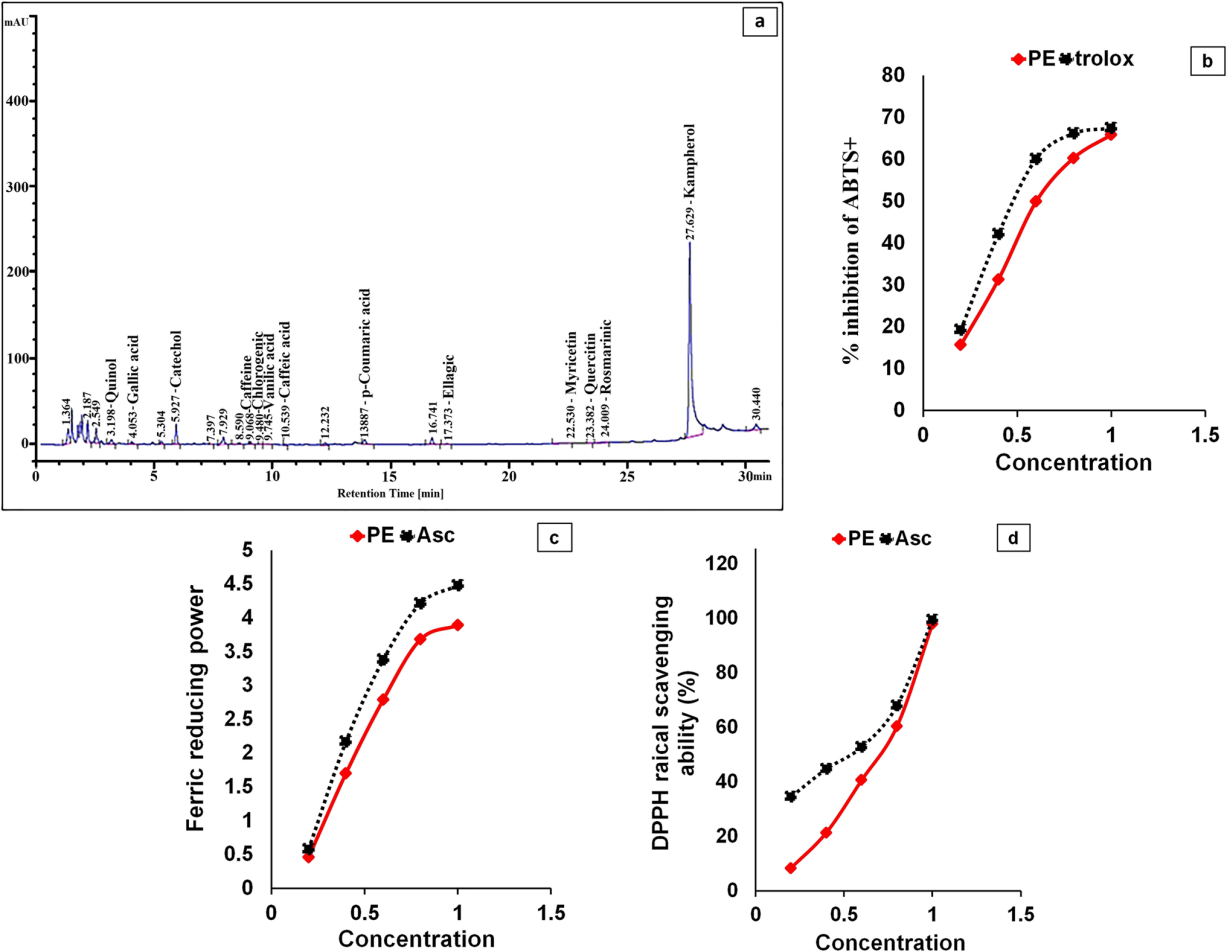
Characterization of the *C. papaya* pulp extract (PE). **a** HPLC chromatogram of PE; **b** 2,2-azino-bis (3-ethylbenzthiazoline-6-sulfonic acid) (ABTS) scavenging activity of PE; **c** ferric-reducing antioxidant power (FRAP) of PE; **d** α, α-diphenyl-β-picrylhydrazyl (DPPH) scavenging activity of PE. Asc and Trolox are standard. Results are shown as mean ± SD (*n* = 3)

The PE contains substantial amounts of phenolics, flavonoids, triterpenoids, tannins, and Asc, according to the phytochemical components (Table 2). HPLC analysis revealed that PE includes a variety of phenolic and flavonoid components (Fig. 2). Table 2 also shows that PE contains a variety of minerals, which were arranged according to their concentration gradients: K ˃ Ca ˃ Na ˃ Mg ˃ S ˃ Fe ˃ Zn ˃ Se ˃ Cu ˃ Mn ˃ Ni ˃ Mo ˃ Co.

**Table 2:** Phytochemicals and minerals ingredients of *C. papaya* pulp extract (PE). All values are presented as mean ± SD (*n* = 3)

Phytochemicals ingredients			
Compound	Concentration (mg eq/g extract)	Compound	Concentration (mg eq/g extract)
Total phenolics	38.79 ± 0.00	Triterpenoids	0.571 ± 0.00
Total flavinoids	7.06 ± 0.00	Ascorbic acid	0.241 ± 0.01
Tannins content	72.84 ± 0.01		
Elements compositions			
Elements name	Concentration (mg/100 g tissue)	Elements name	Concentration (mg/100 g tissue)
K	1157 ± 0.02	Se	1.1 ± 0.00
Ca	557 ± 0.00	Cu	0.94 ± 0.00
Na	269.35 ± 0.00	Mn	0.895 ± 0.00
Mg	248.75 ± 0.00	Ni	0.06 ± 0.00
S	99.15 ± 0.00	Mo	0.04 ± 0.00
Fe	3.545 ± 0.00	Co	0.005 ± 0.00
Zn	1.815 ± 0.00

#### Antioxidant capacities

The TAC of PE was found to be 552.7 mg Asc eq/g. The IC_50_ values for ABTs + and DPPH scavenging activities were calculated and presented in mg/mL. PE’s scavenging activity was indirectly proportional to the IC_50_ values. PE had IC_50_ values of 0.697, 9.262, and 0.601 mg eq/mL for DPPH radical scavenging ability, ferric-reducing power, and anti-ABTs + potential, respectively (Fig. 2). PE and Asc’s antioxidant potentials versus DPPH and ABTs + revealed that PE inhibited ROS in a concentration-dependent style.

#### Prophylactic and curative role of PE against CCl_4_-induced hepatotoxicity

##### PE diminished CCl_4_-induced hepatotoxicity

The administration of rats with CCl_4_ (CCl_4_ group) triggered significantly (*p* < 0.05) declines in TP and albumin levels with significant elevations (*p* < 0.05) in ALT, AST, and ALP activities matched to the C group (Table 3). In contrast, PE treatment pre, during, and/or after CCl_4_ administration enhanced liver functions as shown from the increases of the levels of albumin and TP significantly (*p* < 0.05) compared with the CCl_4_ group, while the activities of ALT, AST, and the ALP were significantly (*p* < 0.05) reduced. Otherwise, the healthy rats which were given PE alone, the liver functions were not significantly altered (*p* > 0.05) when compared to healthy untreated rats (Table 3).

**Table 3:** Effect of PE on liver functions, lipid profile, and kidney functions

Parameters	Groups				
	C	PE	CCl_4_	PE-CCl_4_	PE-CCl_4_-PE
TP (g/dl)	54.3 ± 0.02^*^	54.5 ± 01^*^	5.08 ± 0.00^#^	26.9 ± 0.00	39.7 ± 0.00
Albumin (g/dl)	36.1 ± 0.68^*^	35.5 ± 0.57^*^	5.84 ± 1.60^#^	13.4 ± 0.77	24.9 ± 2.70
ALT (U/L)	16.0 ± 2.13^*^	16.0 ± 2.05^*^	91.0 ± 3.49^#^	46.7 ± 3.92	27.5 ± 4.09
AST (U/L)	44.6 ± 4.15^*^	40.8 ± 3.33^*^	226.9 ± 4.57^#^	113.9 ± 11.47	80.7 ± 2.06
ALP (U/L)	67.2 ± 2.12^*^	72.32 ± 2.86^*^	171.0 ± 4.24^#^	125.9 ± 3.21	115.2 ± 7.01
TG (mg/dl)	112.8 ± 6.08^*^	111.0 ± 9.67^*^	412.2 ± 5.83^#^	312.2 ± 8.65	267.7 ± 5.49
LDL-c (mg/dl)	52.4 ± 0.22^*^	56.0 ± 4.12^*^	831.9 ± 1.82^#^	388.2 ± 3.58	275.9 ± 3.03
HDL-c (mg/dl)	40.6 ± 1.77^*^	38.8 ± 2.31^*^	13.8 ± 2.31^#^	23.1 ± 2.59	30 ± 02.67
S. urea (mg/dl)	22.5 ± 1.73^*^	22.4 ± 1.57^*^	98.9 ± 3.50^#^	54.5 ± 3.43	38.2 ± 0.79
Creatinine (mg/dl)	0.38 ± 0.05^*^	0.37 ± 0.05^*^	1.83 ± 0.08^#^	1.18 ± 0.05	0.96 ± 0.12

All values are presented as mean ± SD (*n* = 8), (^*^) indicates significance when compared to the control at *p* < 0.05, and (^#^) indicates significance when compared to the CCl_4_ group at *p* < 0.05. C group: control rats; PE group: rats received only papaya pulp extract; CCl_4_ group: rats administrated with CCl_4_; PE-CCl_4_ group: rats received papaya pulp extract before and during CCl_4_ injection; PE-CCl_4_-PE group: rats received papaya pulp extract before, during, and after CCl_4_ injection

##### Levels of lipid profile

The administration of rats with CCl_4_ caused significant changes in the lipid profile, where HDL-c level was decreased significantly (*p* < 0.05), while LDL-c and TG levels were elevated significantly (*p* < 0.05), compared with the C group (Table 3). Otherwise, treatment of rats with PE pre, during, and/or following CCl_4_ administration improved the lipid profile where there was a significant (*p* < 0.05) increase in HDL-c level compared to the CCl_4_ group with significant (*p < *0.05) declines in TG and LDL-c levels. Administration of PE alone caused nonsignificant fluctuations in the lipid profile as compared to the C group (Table 3).

##### PE treatment diminished OS in the liver caused by CCl_4_

**Fig. 3:**
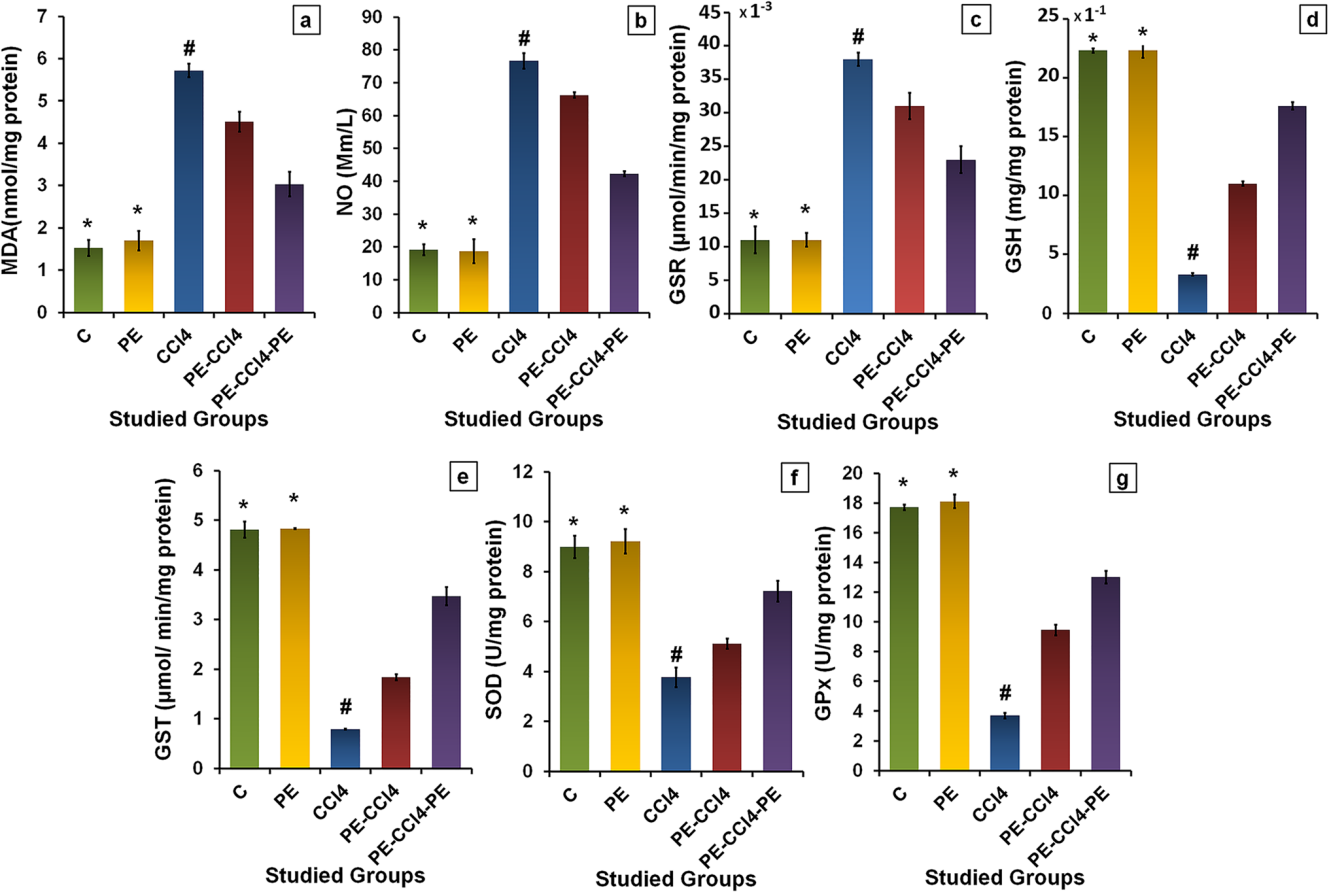
Effect of PE on CCl_4_-induced oxidative stress in the liver. **a** MDA levels; **b** NO levels; **c** GSR activities; **d** GSH levels; **e** GST activities; **f** SOD activities; and **g** GPx activities. Where all values are presented as mean ± SD (*n* = 8), (*) = significance as compared with control at *p* < 0.05, (#) = significance as compared with the CCl_4_ group at *p* < 0.05. C group: control rats; PE group: rats receive only papaya pulp extract; CCl_4_ group: rats administrated with CCl_4_; PE-CCl_4_ group: rats received papaya pulp extract before and during CCl_4_ injection; PE-CCl_4_-PE group: rats received papaya pulp extract before, during, and after CCl_4_ injection

The levels of MDA and NO and GSR activity in rats administered with CCl_4_ were elevated significantly (*p* < 0.05) related to the C group (Fig. 3). However, the GSH level and GST, SOD, and GPx activities were decreased significantly (*p* < 0.05). Treatment with PE pre, during, and/or after CCl_4_ injection significantly (*p* < 0.05) reduced MDA and NO levels and GSR activity as assimilated with the CCl_4_ group. Also, these treatments improved significantly (*p* < 0.05) the GSH level and the activities of GST, t-GPx, and SOD. PE administration to healthy rats exhibited nonsignificant (*p* > 0.05) differences in MDA, NO, and GSH levels and GSR, GST, GPx, and SOD activities equated with the control rats (Fig. 3).

##### PE treatment diminished liver inflammation caused by CCl_4_

**Fig. 4:**
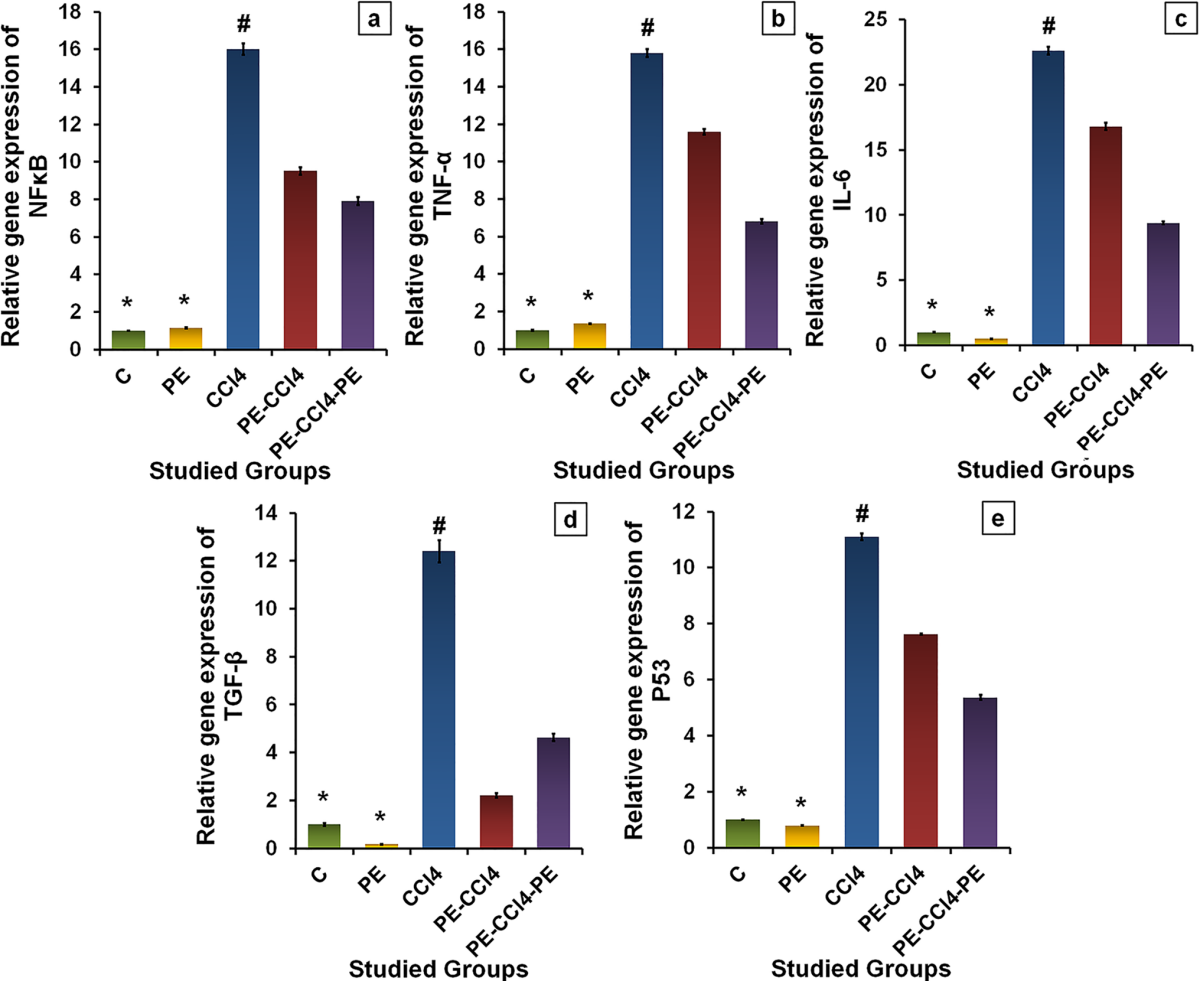
Effect of PE on the hepatic inflammation, fibrosis, and apoptosis stimulated by CCl_4_ administration. The relative gene expression of **a** NF-κB, **b** TNF-α, **c** IL-6, **d** TGF-β, and **e** p53. Where all values are presented as mean ± SD (*n* = 8), (*) = significance as compared with control at *p* < 0.05, (#) = significance as compared with the CCl_4_ group at *p* < 0.05. C group: control rats; PE group: rats receive only papaya pulp extract; CCl_4_ group: rats administrated with CCl_4_; PE-CCl_4_ group: rats received papaya pulp extract before and during CCl_4_ injection; PE-CCl_4_-PE group: rats received papaya pulp extract before, during, and after CCl_4_ injection

The relative gene expressions of NF-κB, IL-6, and TNF-α were up-regulated significantly (*p* < 0.05) in rats after CCl_4_ administration when compared with the C group (Fig. 4a–c). Conversely, their expressions were down-regulated significantly (*p* < 0.05) in rats treated with PE pre, during, and/or after administration of CCl_4_ associated with the CCl_4_ group. Also, administration with PE caused nonsignificant (*p* > 0.05) alterations in the levels of inflammatory as related to the C group (Fig. 4a–c).

##### PE treatment diminished liver fibrosis and apoptosis caused by CCl_4_

The expressions of p53 and TGF-β gene levels were significantly (*p* < 0.05) up-regulated in rats injected with CCl_4_, associated with the C group (Fig. 4d and e), while their expressions were down-regulated significantly (*p* < 0.05) in rats treated with PE pre, during, and/or after CCl_4_ administration as compared with the CCl_4_ group. Further, the levels of TGF-β and p53 gene expressions changed nonsignificantly (*p* > 0.05) in the healthy rats after PE administration compared with the C group (Fig. 4).

##### PE treatment improved renal dysfunction induced by CCl_4_

The CCl_4_ administration caused nephrotoxicity where serum creatinine and urea levels were significantly (*p* < 0.05) increased compared to the C group. Conversely, PE treatment pre, during, and/or after CCl_4_ administration reduced nephrotoxicity as exposed from the significant (*p* < 0.05) reduction of urea and creatinine levels. The renal functions changed nonsignificantly (*p* > 0.05) in healthy rats after PE treatment (Table 3).

##### Liver histopathology of different studied groups

**Fig. 5:**
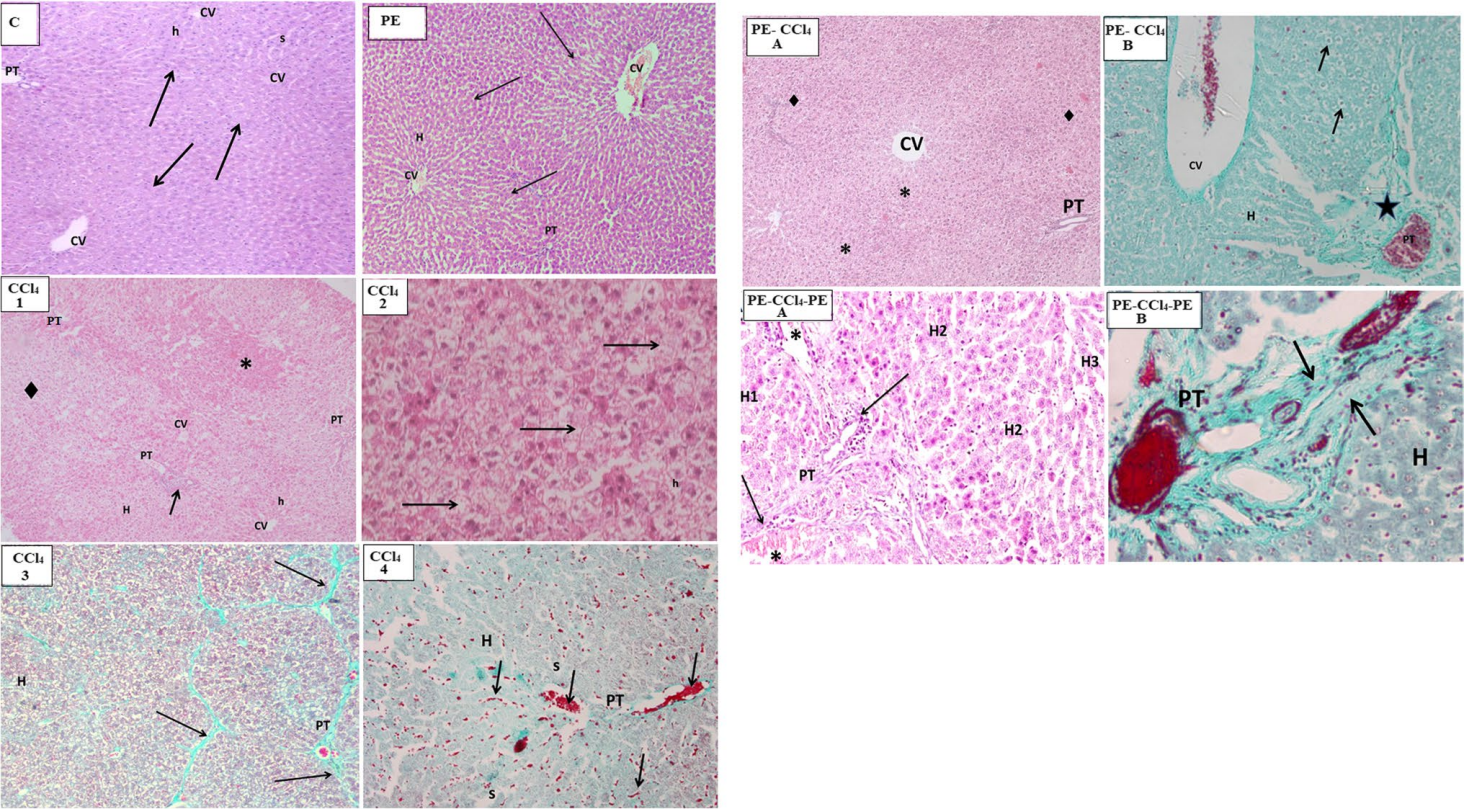
Microscopic examination of rat liver tissues from different groups. Where (C) represents the control group (H & E stain, Mic. Mag. × 100). This shape shows that the hepatic lobules (arrows) were arranged in a normal organization, the cords of hepatocytes (h) which radiate from the central veins (CV) are separated by narrow slit-like sinusoids (s), and a normal portal tract (PT) is demonstrated at the upper left corner of a hepatic lobule. (PE) signifies the PE group (H & E stain, Mic. Mag. × 100): normal liver architecture was demonstrated, and the central veins (CV) occupied the center of the hepatic lobule (arrows) with cords of hepatocytes (H) radiating from them, and the portal tract (PT) was located at the angle between the adjacent hepatic lobules. [CCl_4_ (1) and (2)] represent the CCl_4_ group stained with H & E. (1) [Mic. Mag. × 100] shows focal areas of eosinophilic hepatocytes (*) alternate with pale areas of vacuolated hepatocytes (♦), a preserved organization of the cords of hepatocytes (H) in many hepatic lobules was noticed, and some portal tracts (PT) show dense cellular infiltration (arrow). In (2) (Mic. Mag. × 400), diffused vacuolation of hepatocytes (h) and groups of degenerated hepatocytes without nuclei (arrow) have appeared. [CCl_4_ (3) and (4)] signifies the CCl_4_ group stained with Gomori’s trichrome stain and Mic. Mag. × 100. In (3), thick green bands of fibers were noticed between the liver lobules (arrows), and the vacuolated hepatocytes also appeared. In (4), dilatation of blood sinusoids (s) between hepatocytes (h) and the abnormal inspissation of red blood cells (arrows) in the hepatic sinusoids as in the portal tract tributaries (PT) were presented. The (PE-CCl_4_) represents the microscopic examination of rat liver tissue of the PE-CCl_4_ group. In **A** (H & E stain, and Mic. Mag. × 100), the histological changes persisted as foci of intact eosinophilic hepatocytes (♦) adjacent to pale vacuolated hepatocytes (*). CV, central vein; PT, portal tract. However, in **B** (Gomori’s Trichrome stain and Mic. Mag. × 400), excessive collagen (star) depositions appeared in the portal tract (PT), and a wide dispersion of degenerated cells (arrow) was noticed among the hepatocytes (H). CV, central vein with inspissated blood. The (PE-CCl4-PE) denotes the PE-CCl_4_-PE group. In **A** (H & E stain, and Mic. Mag. × 100), partial recovery varied between apparently normal hepatocytes (H1), vacuolated hepatocytes (H2), and thinned-out hepatic cords formed of degenerated dense cells with dark nuclei (H3). The distorted structure of the portal tract (PT) with persistent excessive stromal cellular infiltrates (arrow) and dilated, blood-engorged portal vein tributaries (*) was illustrated. In **B** (Gomori’s Trichrome stain, Mic. Mag. × 400), the abundant deposits of collagen fibers were illustrated (arrows pointing to green bands). “H,” hepatocytes

Histopathological examination of the C group showed normal histological structure of the liver with no remarkable pathological changes (Fig. 5, C). The results of the healthy rats after PE administration (Fig. 5, PE) displayed that there are no variations in the liver histology when equated with the C group indicating that the natural phyto-antioxidants of PE did not induce any apparent alterations neither in the hepatic parenchyma (liver cells) nor in the stroma (connective tissue content). CCl_4_ administration induced dispersed focal degenerative changes in the liver parenchyma appeared as focal pale areas with hepatocyte vacuolation (steatosis) or cell degeneration (empty cells with dark pyknotic nuclei) alternating with foci with intact eosinophilic hepatocytes (Fig. 5, CCl_4_ 1). A histopathological feature described as piece meal degeneration. On the level of individual cells (Fig. 5, CCl_4_ 2), groups of pale degenerated hepatocytes were seen with individual intact eosinophilc hepatocytes in between. CCl_4_ affected also the liver stroma as it enhanced the deposition of abundant bundles of collagen fibers in the portal tract, around the central veins and along the boundaries separating between the liver lobules resulting in distortion of general architecture of lobules. It was also associated with hemorrhages in the micro-vasculature of the liver (central vein, hepatic sinusoids, and portal tract vessels) (Fig. 5, CCl_4_ 3 and 4). On the contrary, PE treatment before and during CCl_4_ injection (Fig. 5, PE-CCl_4_, A and B) and PE treatment before, during, and after CCl_4_ injection (Fig. 5, PE-CCl_4_-PE, A and B) caused a relative improvement in the histopathology of the hepatocyte lesion caused by CCl_4_, since the last treatment gave the best results.

## Discussion

The CCl_4_ continues to serve as a valuable pattern chemical for understanding the mechanisms of hepatotoxic consequences such as fatty degeneration, fibrosis, cirrhosis, and carcinogenicity (Saile and Ramadori 2007; Diao et al. 2011). Otherwise, liver disease’s incidence is increasing worldwide due to the uses of drugs, chemical poisons, viral infections, and alcohol intake. Previous studies showed that the antioxidants from plant origin perform a crucial role in the detoxification ensued from CCl_4_. Therefore, in this study, we illuminated the mechanism of hepatotoxicity induced by CCl_4_ via generating of active metabolites and other free radicals. In addition, we evaluated the role of PE components in the liver protection and detoxification of hepatotoxicity and improving liver functions and liver architecture. The results of this study indicated that the administration of CCl_4_ resulted in a significant increase in the levels of MDA (a lipid peroxidation product) and NO (a highly reactive molecule, and the main product of RNS) and GSR activity relative to the C group. However, there were a reduction in GSH level and the activities of SOD, t-GPx, and GST in comparison with the C group. This suggests that CCl_4_ increased the OS, which lead to the elevation of lipid peroxidation of polyunsaturated fatty acids and oxidation of protein and other macromolecules and this led to liver damage.

Mechanistic studies demonstrated that the metabolism of CCl_4_ via CYP2E1, to hugely reactive free radical (CCl_3_• and CCl_3_OO•), performs a crucial role in the presumed style of action. As CCl_3_• and CCl_3_OO• can covalently bind locally to cell macromolecules, priority is given to the polyunsaturated fatty acids of the membranes where the free radicals trigger lipid peroxidation by assaulting polyunsaturated fatty acids causing generation of chain of free radicals (Zhao et al. 2017; Shaban et al. 2021c). The peroxidation of lipid membrane causes its disruption, and this interrupts the permeabilities of mitochondrial, endoplasmic reticulum, and plasma membranes, leading to the harm of membrane probity, loss of calcium cell detention, and homeostasis. All these changes can make a significant contribution to subsequent cell damage and leakage of microsomal enzymes (Shaban et al. 2013; Zhao et al. 2017). Reactive aldehydes, particularly 4-hydroxynonenal, are fatty acid breakdown products that bind readily to protein functional groups and impede the activity of key enzymes. Additionally, the elevation of the NO level after CCl_4_ administration indicating that CCl_4_, CCl_3_•, and CCl_3_OO• activated the inducible nitric oxide synthase (Germoush et al. 2018; Munakarmi et al. 2020) where the elevation in the NO level suppressed the growth of the lymphocytes and injures the encirclement cells (Munakarmi et al. 2020; Shaban et al. 2021a, b, c). Previous research has also shown that CCl_4_ poisoning causes hypomethylation of cellular components, which inhibits protein synthesis in RNA and lipoprotein secretion in phospholipids (Dalle-Donne et al. 2009).

In addition, nonenzymatic antioxidant GSH protects cells from the damaging effects of reactive oxygen species. The GSH can neutralize and scavenge the free radicals, where the GSH is oxidized to GSSG (Shaban et al. 2013, 2014, 2022a, b, c; Habashy et al. 2018). Also, in the OS state and via protein S-glutathionylation, GSH participates in the preservation of the thiol and organization of the thiol proteins redox in the cells (Shaban et al. 2013, 2014) according to the reaction:

$$\mathrm{RSH}+\mathrm{GSH}+\left[\mathrm{O}\right]\to \mathrm{GSSR}+{\mathrm{H}}_{2}\mathrm{O}$$

Furthermore, GSH is utilized to detoxify hazardous compounds such as methylglyoxal and formaldehyde, which are produced as a result of the OS, via the glyoxalase system (glyoxalase I and II) (Dringen et al. 2015). Likewise, GSH is important as a cofactor for the GPx, a selenoprotein enzyme, which reduces inorganic and organic hydroperoxides (Shaban et al. 2014; Germoush et al. 2018). Also, GSH is used as a substrate for GST, a drug-metabolizing enzyme. When this enzyme reacts with numerous dangerous chemical species like halides, epoxides, and free radicals, it helps create inactive products (Shaban et al. 2014). Additionally, GSH plays an important role in the reduction of methemoglobin (MetHbFe^+3^) into hemoglobin (HbFe^+2^), but in proteins, it performs in the creation and maintenance of disulfide bonds (Dorman et al. 2002). Therefore, all these reactions of GSH led to reduction its level in rats after CCl_4_ administration. Moreover, the decline in GSH level may be owed to its reaction with NO or ONOO − to produce S-nitroso GSH (Van der Vliet et al. 1997). Wherever, GSH reduction could also contribute to the stimulation of lipid peroxidation (Shaban et al. 2013). In contrast, GSR is responsible for the conversion of GSSG to GSH to maintain the redox state in the cells. The elevation of GSR activity after CCl_4_ administration perhaps suggests an alteration to oxidative condition (Korhonen et al. 2005). The inhibition of GPx and GST activities in rats after CCl_4_ administration may be owed to the reduction of GSH level. Also, their inhibition could be associated with the direct communication of the free radicals such as CCl_3_•, CCl3OO•, and reactive aldehydes with the functional groups of these enzymes. Otherwise, SOD catalyzes the diversion of the superoxide radical (O_2_•¯) into hydrogen peroxide (H_2_O_2_) (Shaban et al. 2014, 2013). The inhibition of SOD in rats after CCl_4_ administration, in this study, may be due to the interaction of the free radicals with its active site or with the enzyme gene expression (Shaban et al. 2013, 2022c).

At the molecular level, our findings revealed that CCl_4_ administration triggered up-regulation of the gene expressions of NF-κB, TNF-α, TGF-β, IL-6, and p53 indicating that CCl_4_ activated these markers. The NF-κB, a transcription factor, performs a main function in the progressions of inflammation and apoptosis. NF-κB is stimulated by a diversity of inducers, involving inflammatory cytokines among cells, and pathogen-originated materials (Abdel-Rahman et al. 2016; Chen et al. 2018). Normally, NF-κB exists in the cytoplasm in the deactivated form since it is combined with IκBα, an inhibitory subunit. While throughout the OS state, the ROS induces phosphorylation leading to the separation of the IκBα subunit resulting in activation of NF-κB. The activated NF-κB leakages into the nucleus and induces the expression of inflammatory mediators (Girard et al. 2009; Chen et al. 2018). So, the stimulation of NF-κB gene expression in this study revealed that CCl_4_ provoked liver inflammation. Otherwise, the stimulation of gene expression of IL-6, TNF-α, and TGF-β, beside elevation of the NO level, indicates that CCl_4_ induced fibrosis and apoptosis. TNF-α is a pro-inflammatory cytokine that interferes with liver damage through a variety of biological functions (Dinarello 2000). Also, IL-6, a pro-inflammatory mediator released by Kupffer cells (KCs), stimulates the biosynthesis of the cytokines which participates in the inflammatory response of the induced liver damage (Dinarello 2000). Also, TGF-β is a key mediator for the progression of inflammatory response and fibrosis. TGF-β regulates the inflammation and fibrosis through the interaction with a NF-κB pathway. TGF signaling and hepatic stellate cells (HSCs) are both activated by active NF-κB, and the activated HSCs are changed into myofibroblasts, which promote collagen deposition in the extracellular matrix (Meyer et al. 1990; Eltahir et al. 2020). Moreover, p53 is a well-known tumor suppressor protein that manages DNA repair systems and the cell cycle seizure in cases of prolonged OS exposure, mitogenic oncogenes, apoptosis, etc. (Han et al. 2019). Our data showed up-regulation of p53 gene expression in hepatocytes of rats administered with CCl_4_ and this indicates that CCl_4_ induced apoptosis that increased with increasing of the OS. Also, the stimulation of a NF-κB pathway leads to up-regulation of p53 gene expression (Lee et al. 2019). Otherwise, the elevation of NO in rat hepatocytes after CCl4 administration suggests that CCl_4_ caused apoptosis since NO may react with O· − 2 radicals producing the peroxynitrite anion (ONOO −) resulting in DNA damage and stimulation of the nuclear poly-ADP-ribose polymerase (PARP-1). PARP-1 motivates NAD + hydrolysis, which results in the cellular energy depletion and necrotic cell death (Saada et al. 2010). Moreover, accumulation of NO· in mitochondrial leads to the depolarization of mitochondrial and leaks the cytochrome c from mitochondria to the cytosol causes apoptosis (López et al. 2010). Moreover, the elevation of OS promotes apoptosis via up-regulation of gene expression of Baxand p53 and down-regulation of Bcl-2 and Bcl-xL gene expression (Han et al. 2019).

Otherwise, the histopathological examination confirmed the biochemical and molecular results since histopathology of the rat liver after CCl4 administration showed extensive histological modifications in the hepatic tissues; characterized by severe hepatocellular deteriorations, necrosis, fatty alterations, and existence of inflammatory cells. Therefore, our data showed the levels of serum AST, ALT, and ALP were elevated after CCl_4_ administration as matched with the C group, while albumin and TP levels were declined. This established that CCl_4_ induced liver injury which leads to decline the protein biosynthesis and leakage of the liver enzymes into the blood circulation. Also, CCl_4_ poisoning causes hypomethylation of cellular modules, which suppresses protein synthesis in the case of RNA (Unsal et al. 2021). Additionally, the data exposed that CCl_4_ intoxication changed the lipid profile, where LDL-c and TG levels were raised but HDL-c level was dropped. This may be related to the liver damage and the failure of liver cells to metabolize lipid, besides impairing the transformation of cholesterol to bile acids. Moreover, CCl_4_ administration caused significant elevations in creatinine and urea levels, as matched to the C group indicating that CCl_4_ induced nephrotoxicity. Our conclusions concur with the previous findings, which described that CCl_4_ caused hepatotoxicity and nephrotoxicity (Shaban et al. 2021a, b, c, 2022a, b).

On the other hand, in this study, the liver pathohistological outcomes confirmed that therapy of rats with PE before, throughout, and/or following CCl_4_ administration diminished the hepatic injury created by CCl_4_ and improved the liver architecture. Consequently, the liver functions and lipid profile were improved significantly as shown from the reduction of AST, ALT, ALP, TG, and LDL-c with elevations in the total protein and HDL-c levels when contrasted with the CCl_4_ group. Also, the attenuation of the liver injury induced by CCl_4_ was proven by the diminution of the OS, inflammation, fibrosis, and apoptosis as revealed from the results which we discussed as follows. The current data revealed that PE treatment reduced the OS as NO and MDA levels and the activity of GSR were decreased as matched by the CCl_4_ group, while the activities of GST, SOD, and t-GPx and GSH level were increased. The reduction in OS designates that PE has antioxidant activity against CCl_4_ intoxication and has capable scavenging activities against ROS and RNS. Our data revealed that PE is rich in phenolic compounds, flavonoids, tannins, triterpenoids, and Asc. Moreover, PE analysis using HPLC revealed that it contains quinol, caffeine, chlorgenic acid, caffeic acid, vanillic acid, ellagic acid, myricetin, and rosmarinic acid. Additionally, previous studies elucidated that PE contains phytosterols and tocopherols (Rodrigues et al. 2019). All these compounds as well as some minerals in PE, especially Zn, Se, Cu, Mn, and Ni (Table 2), have antioxidant activities against ROS and RNS (Alotaibi et al. 2017; Shaban et al. 2013). In the cells, these compounds exhibit the protection and therapeutic effects against oxidative damage, but with different mechanisms, some of them were discussed later. The antioxidant activity of PE was proved with the current results which revealed that the TAC of PE in vitro is extremely high. Also, PE has scavenging activities against ABTS + and DPPH and ferric-reducing power. As a result, the reduction of NO and MDA in rats treated with PE could be linked to the polyphenolic compounds in PE where polyphenolics are excellent inhibitors for the nitrosation process and can prevent oxidative damage due to their ability to scavenge ROS and RNS. Also, polyphenolic substances boost GSH levels as well as the activities of t-GPx and SOD, but they limit GSR activity (Moskaug et al. 2005; Shaban et al. 2013). The antioxidant abilities of plant polyphenols have been linked to their reactivity as electron or hydrogen donors, ability to stabilize unpaired electrons, and ability to terminate Fenton processes (Shaban et al. 2013; Eltahir et al. 2020). The mechanism of the phenolics action as an antioxidant differs according to their structures where chlorogenic reacts with free radicals producing new radicals, which are stabilized by the action of electron resonance of the aromatic nucleus in its structure (Jung et al. 1999; Shaban et al. 2013), while vanillin reacts with the free radicals via self-dimerization (Tai et al. 2012). In contrast, vanillic acid has moderate antioxidant and anti-inflammatory activities because its carboxyl group acts as an electron donor subunit or self-dimerization with the free radicals (Vinoth and Kowsalya 2018). GA interferes with ROS generation (Bello and Idris 2018). However, in the case of quinols, the quinol group (QH2) interacts with the peroxyl radical (ROO•) forming semiquinone radical (QH•) which can reduce another ROO• since it has been shown that the interaction between ROO• radical with quinols is faster than its interaction with lipid molecules. This process leads to quench ROO• resulting in the prevention of the formation of more radicals as lipid peroxyl (LOO•) and terminates the lipid peroxidation process (Lokhmatikov et al. 2014; Shaban et al. 2021a, b, c). Catechol molecule and catechol-containing flavonoids such as quercetin have a nonenzymatic antiradical scavenging activity. During the OS, catechol moiety changes to semiquinone radicals and quinones by oxidation where the oxidized products can arylate the critical free SH group of GSR and inhibit its enzyme activity (Boots et al. 2003). Furthermore, ellagic acid reduces CCl_4_ metabolism via the reduction of total hepatic CYP2E1 and CYP-450 (Ahn et al. 1996; Shaban et al. 2014), while caffeine and its metabolite (theophylline) are potent inhibitors against HO•¯via its trapping (Vieira et al. 2020). Rutin, myricetin, and ellagic acid inhibit xanthine oxidase activity leading to the suppression of the O2•¯ formation resulting in the increase in SOD activity (Zhang et al. 2017). However, kaempferol activates the production of antioxidant enzymes like catalase, GPx, and GST (Yang et al. 2014). In general, polyphenolics boost GSH levels and t-GPx and SOD activities, but they suppress the GSR activity (Moskaug et al. 2005; Shaban et al. 2013). Because of their ability to scavenge ROS and RNS, polyphenolics, notably chlorogenic acid and caffeic acid, are excellent nitrosation inhibitors and can prevent oxidative damage. Polyphenols may thus be effective not only in reducing oxidative damage but also in inhibiting the formation of mutagenic and carcinogenic n-nitroso compounds in the body (Shaban et al. 2014). Moreover, hydroxycinnamic acids, including coumaric and caffeic, are effective antioxidants through the donation of electrons or hydrogen atoms, and this attached to the presence of a phenolic nucleus and the side chains (Liu et al. 2020). Otherwise, rutin and tannins exhibit antioxidant properties by chelating metal ions, for example, Fe (II), stopping the Fenton process, and thereby ending OS (Ahn et al. 1996; Karamać 2009; Saravanan et al. 2006; Shaban et al. 2013 and 2014). As well, the incidence of Asc and triterpenoids in PE increases its antioxidant power, whereas triterpenoids can chelate Fe (II) (Shaban et al. 2014).

Otherwise, our data revealed that PE contains considerable amounts of minerals with different concentrations. The existence of S, Cu, Zn, Mn, and Se in PE stimulates the antioxidant system via the activation of SOD and t-GPX. SOD is found in three isoforms, including (Cu/Zn)-SOD, (Mn)-SOD, and extracellular-SOD; therefore, Cu, Zn, and Mn are essential elements in its activity (Abu-Serie et al. 2018). Also, Se is also involved in the production of t-GPX protein and its function (Bermingham et al. 2014).

Furthermore, the present findings showed that there was a decline in the hepatic TNF-α, NF-κB, IL-6, p53, and TGF-β gene expression, with a significant decline in the NO level in rats injected with CCl_4_ and treated with PE (PE-CCl_4_) and (PE-CCl_4_-PE), when compared with the CCl_4_ group. This signifies that PE has anti-inflammatory, antifibrotic, and antiapoptotic influences and this is because of its valuable components mentioned above, especially phenolics and flavonoids. Myricetin and kaempferol in PE besides their actions as antioxidants inhibit the effect of several cytokines like TNF-α, IFN-γ, IL-2, and IL-6, demonstrating their anti-inflammatory and antifibrotic roles (Cao et al. 2014; Sekiguchi et al. 2019). Also, previous investigations revealed that kaempferol has antiapoptotic activity (Sekiguchi et al. 2019). Additionally, myricetin reduces NF-κB gene expression and prevents its activation and inhibits the inflammatory markers, especially inducible nitric oxide synthase (iNOS) and cyclooxygenase‐2 (Li et al. 2019; Afroze et al. 2020). Moreover, treatment with PE reduced the nephrotoxicity induced by CCl_4_ as creatinine and urea levels were lower than the CCl_4_ group. This implies that PE has prophylactic and therapeutic purposes towards the kidney toxicity induced by CCl_4_. Generally, our results showed that phenolic and flavonoid compounds, terpenoids, Asc, some minerals, etc., in PE performed a critical role in reducing the free radicals generated from CCl_4_ metabolism. And this led to the reduction of lipid peroxidation and safeguarding membrane lipids from the oxidative destruction, inflammation, fibrosis, and apoptosis. Commonly, the results showed that PE treatment (pre, during, and after CCl_4_ administration) gives better results than the treatment with PE pre and during CCl_4_ administration.

Otherwise, the present outcomes confirmed that administration of healthy rats with PE alone for 14 weeks triggered nonsignificant alterations in markers of the OS, inflammation, and apoptosis as compared to the C group, while there were no variations in liver histology.

## Conclusion

**Fig. 6:**
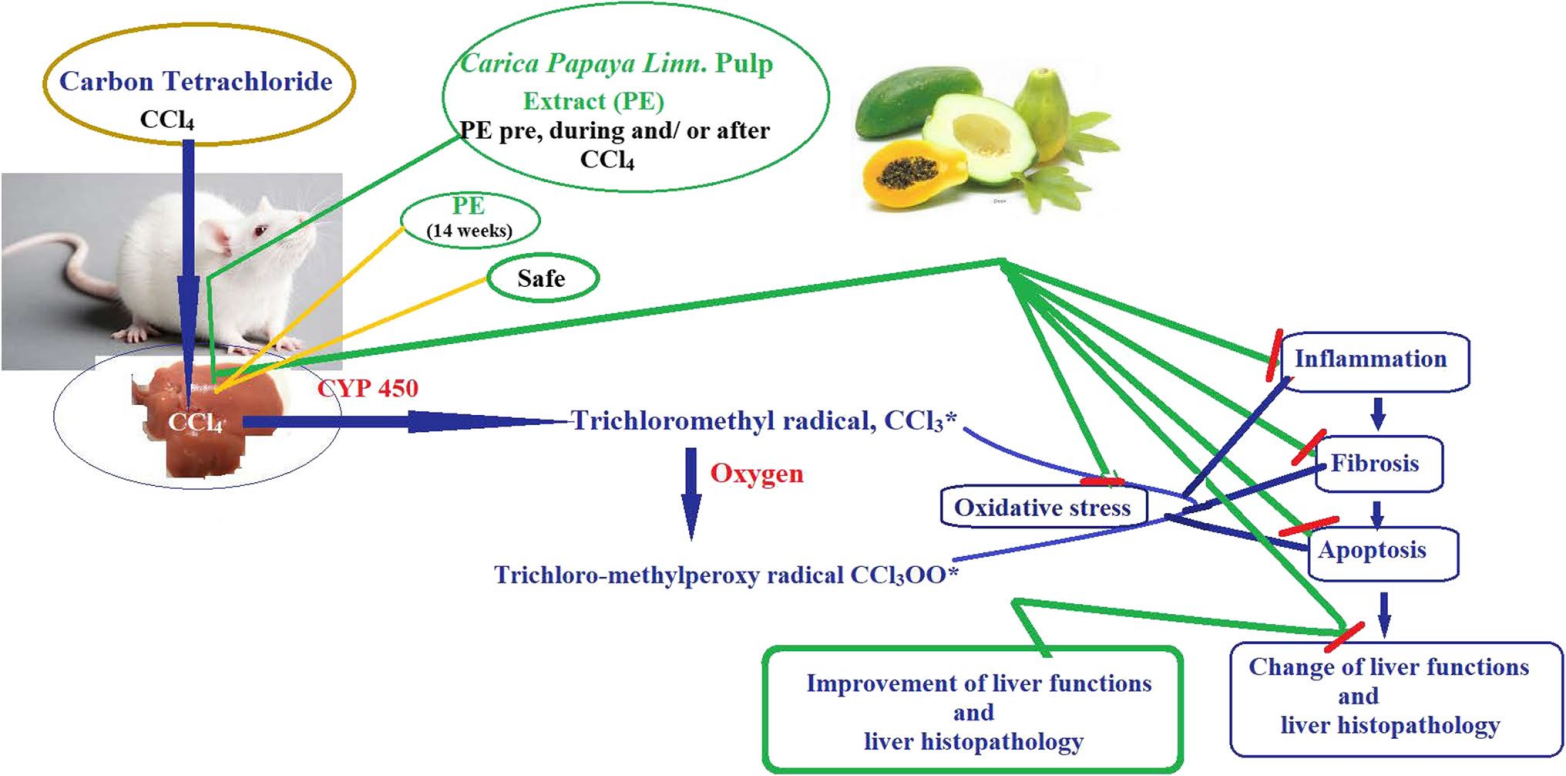
Diagrammatic representation of the protective and therapeutic roles of PE against CCl4-induced rat hepatotoxicity. PE reduced the OS, inflammation, fibrosis, and apoptosis caused by CCl4, leading to improved liver architecture and liver functions

PE demonstrated its protective and therapeutic effect in opposition to hepatotoxicity caused by CCl_4_ by reducing the oxidative stress, infammation, fibrosis, and apoptosis. Additionally, PE treatment diminished the nephrotoxicity induced by CCl_4_ administration. PE treatment reduced PE treatment (pre, during, and after CCl4 administration) gave better results than the treatment with PE pre and during CCl4 administration. The beneficial effect of PE may be due to its content, as it contains high quantities of phenolics, flavonoids, tannins, terpenoids, and ascorbic acid. For 12 weeks, healthy rats were given PE; there were no adverse effects. The prevention and treatment of the toxicity brought on by xenobiotics can thus be accomplished with PE, which is a promising drug. Figure 6 shows the diagrammatic representation of the protective and therapeutic roles of PE against CCl_4_-induced rat hepatotoxicity.

## Data Availability

All data generated or analyzed during this study are included in this published article.

## Abbreviations

PE: *Carica Papaya Linn.* Pulp aqueous extract
ABTS: 2,2-Azinobis(3-ethylbenzothiazoline-6 sulfonic acid)
ALP: Alkaline Phosphatase
ALT: Alanine aminotransferase
AST: Aspartate aminotransferase
BHT: Butylated hydroxytoluene
CCl4: Carbon tetrachloride
DNPH: 2,4 Dinitrophenyl hydrazine
DPPH: 2,2 Diphenyl-1-picrylhydrazyl
GA: Gallic acid
GPx: Glutathione peroxidase
GSH: Glutathione
GSR: Glutathione reductase
GST: Glutathione-S-transferase
HPLC: High-performance liquid chromatography
ICP-OES: Inductively 5100 coupled plasma optical emission spectrometer
IL-6: Interleukin-6
LDL-c: Low-density lipoprotein cholesterol
MDA: Malondialdehyde
NF-κB: Nuclear factor kappa B
OS: Oxidative stress
qRT-PCR: Quantitative real-time polymerase chain reaction
RU: Rutin
SOD: Superoxide dismutase
TBA: Thiobarbituric acid
TG: Triglycerides
TGF-β: Transforming growth factor-β
TNF-α: Tumor necrosis factor-α
TP: Total protein
